# Dielectric Resonance-Based Optical Metasurfaces: From Fundamentals to Applications

**DOI:** 10.1016/j.isci.2020.101868

**Published:** 2020-11-26

**Authors:** Wenwei Liu, Zhancheng Li, Hua Cheng, Shuqi Chen

**Affiliations:** 1 The Key Laboratory of Weak Light Nonlinear Photonics, Ministry of Education, School of Physics and TEDA Institute of Applied Physics, Nankai University, Tianjin 300071, China; 2 The Collaborative Innovation Center of Extreme Optics, Shanxi University, Taiyuan, Shanxi 030006, China; 3 Collaborative Innovation Center of Light Manipulations and Applications, Shandong Normal University, Jinan 250358, China

**Keywords:** Optics, Electromagnetic Waves, Metamaterials

## Abstract

Optical metasurface as a booming research field has put forward profound progress in optics and photonics. Compared with metallic-based components, which suffer from significant thermal loss and low efficiency, high-index all-dielectric nanostructures can readily combine electric and magnetic Mie resonances together, leading to efficient manipulation of optical properties such as amplitude, phase, polarization, chirality, and anisotropy. These advances have enabled tremendous developments in practical photonic devices that can confine and guide light at the nanoscale. Here we review the recent development of local electromagnetic resonances such as Mie-type scattering, bound states in the continuum, Fano resonances, and anapole resonances in dielectric metasurfaces and summarize the fundamental principles of dielectric resonances. We discuss the recent research frontiers in dielectric metasurfaces including wavefront-shaping, metalenses, multifunctional and computational approaches. We review the strategies and methods to realize the dynamic tuning of dielectric metasurfaces. Finally, we conclude with an outlook on the challenges and prospects of dielectric metasurfaces.

## Graphical Abstract

**Figure:**
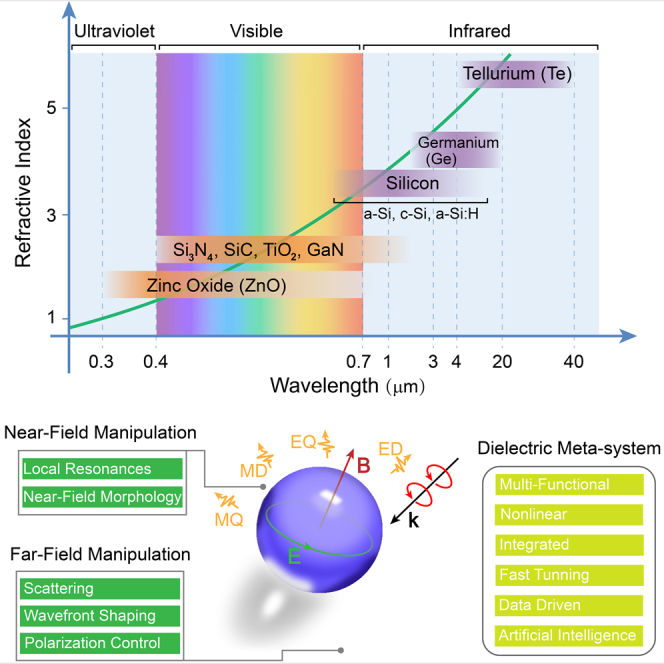

## Introduction

Optical metasurfaces, as a two-dimensional counterpart of metamaterials, have garnered much attention in the scientific community due to the versatile capabilities to manipulate electromagnetic (EM) waves within an ultrathin surface (Chen et al., 2016; Genevet et al., 2017; Luo, 2019; Monticone and Alù, 2017; Neshev and Aharonovich, 2018). The subwavelength nanostructures can interact strongly with the incident light, leading to striking local optical resonances. For example, electric dipole (ED) and magnetic dipole (MD) resonances can be generated with surface currents and current loops (Kim et al., 2018; Liu et al., 2015b; Mutlu et al., 2012; Wei et al., 2011). These local resonances can induce changes in different optical dimensions, such as phase and polarization. By introducing a phase-abrupt, metasurface can manipulate the EM wavefront according to the generalized Snell's law (Arbabi et al., 2017; Yu and Capasso, 2014; Yu et al., 2011). Besides the local resonances, geometric symmetry also provides a basic restriction on the properties of nanostructures. For example, for nanostructures with specific symmetry being able to locally change the polarization state of the incident light, Pancharatnam-Berry (P-B) phase that is proportional to the orientation angles of the nanostructures can be generated for left-handed circularly polarized (LCP) and right-handed circularly polarized (RCP) light (Bomzon et al., 2001; Chen et al., 2020a; Ding et al., 2015; Huang et al., 2012; Li et al., 2017c). Although the P-B phase originates from the space-variant polarization conversion along the Poincaré sphere, the applications of P-B phase in metasurfaces also require local EM resonances to realize applicable efficiency. As a result, the P-B-phase-based nanostructure serves as a local polarization converter, such as a local wave plate (Lin et al., 2014). Metasurfaces provide a versatile platform to engineer the EM waves with a high degree of freedom, such as manipulating the chirality (Chen et al., 2018b; Zhang et al., 2017; Zhu et al., 2018), coherence (Chen et al., 2020b), and topology (Gomez-Diaz et al., 2015; Hu et al., 2018; Mann et al., 2020) of light. Recently, researchers also developed a method to directly shape the near-field landscapes of EM waves from far-field with metasurfaces in the microwave region (Ginis et al., 2020). Based on the efficient manipulation of optical fields with nanostructures, metasurfaces have also been demonstrated to be a promising platform for imaging systems (Aieta et al., 2012; Engelberg and Levy, 2020), holography (Huang et al., 2015; Wen et al., 2015), and quantum applications (Bekenstein et al., 2020; Stav et al., 2018).

**Figure 1:**
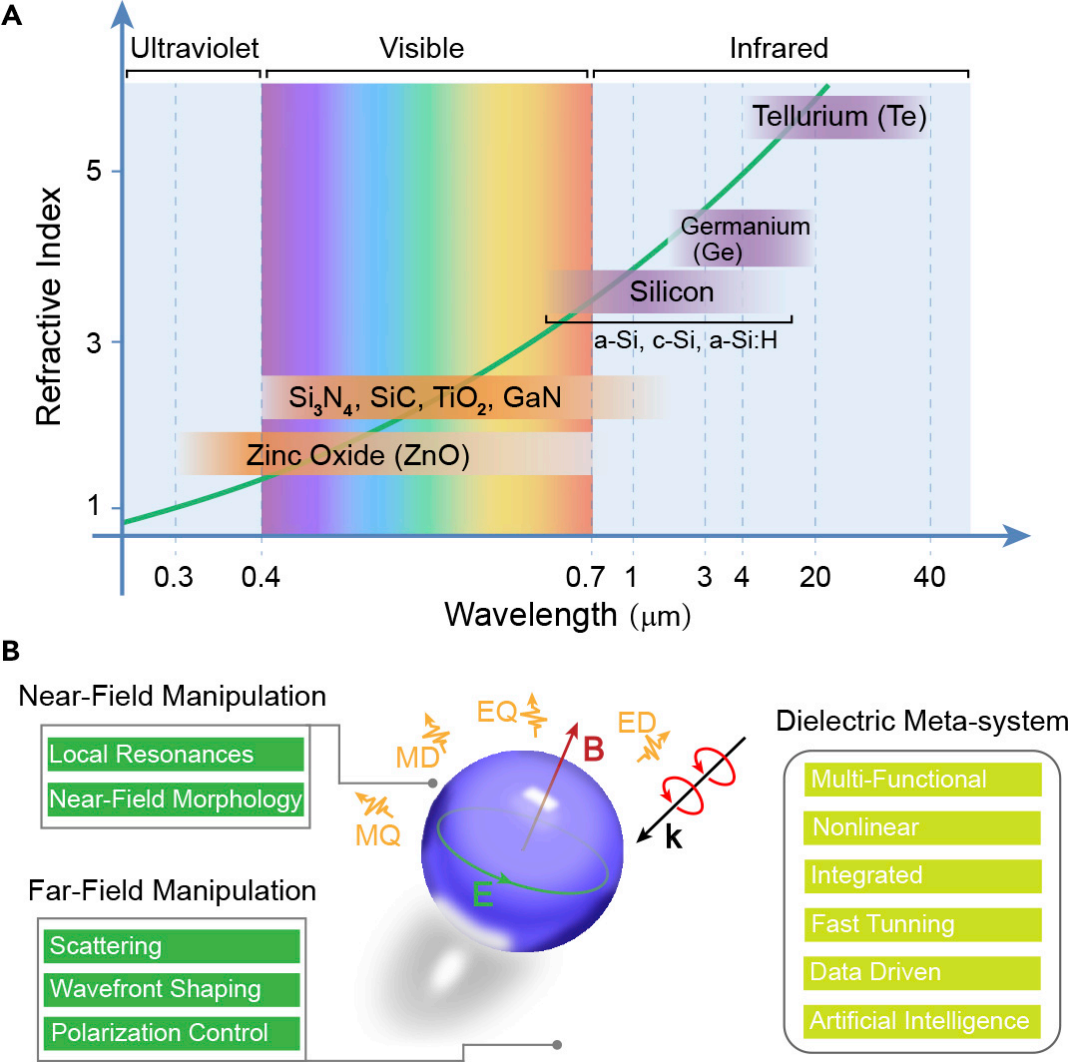
High-Index Dielectric Metasurfaces for Electromagnetic Control (A) Commonly used high-index materials as the building blocks of dielectric metasurfaces from the UV to the infrared wavebands with different refractive indices. (B) The high-index dielectric resonators can realize near-field and far-field manipulation of EM waves with a high degree of freedom, which enables high-performance dielectric meta-systems.

Although the geometric parameters play an important role in artificial photonics, the properties of nanostructures are also fundamentally decided by the building materials. For example, the intrinsic thermal loss in metallic nanostructures often limits the working efficiency of metasurfaces (Karimi et al., 2014; Luo et al., 2015; Yu et al., 2012). High-efficiency metallic metasurface designs usually require more complex designs such as layered structures, taking advantage of the high Q factor of Fabry-Pérot resonances (Grady et al., 2013; Liu et al., 2015a; Minovich et al., 2017; Roth et al., 2020). On the other hand, metallic components are challenging to simultaneously support electric and magnetic resonances within a single nanostructure. In contrast, dielectric building blocks enables simultaneous manipulation of electric and magnetic multipole resonances with negligible thermal loss in the operating waveband (Devlin et al., 2016; Jahani and Jacob, 2016; Li et al., 2015b; Lin et al., 2014), which makes dielectric component a promising candidate for efficient optical and photonic devices. Furthermore, dielectric metasurfaces are also promising to serve as an integrated and on-chip photonic platform. Figure 1A depicts the typical high-index dielectric materials to build artificial nanostructures from the UV to the infrared regions. Based on the remarkable capability of nanostructures to manipulate EM waves, future dielectric meta-system that combines different functions into a single photonic platform may be possible.

In this review, we summarize the recent research hotspots and challenges in dielectric metasurfaces. We make a brief introduction on the concepts and principles of dielectric local resonances, including high Q factor resonances and nonlinear resonances. We review the applications of dielectric metasurfaces such as wavefront control, dielectric metalenses, multifunctional metasurfaces, holography metasurfaces, and computational metasurfaces. We also introduce the current progress on dynamically tuning of dielectric metasurfaces and the basic method and strategy to realize tunable devices. Finally, we present an outlook including the challenges and future developments to guide future investigations of dielectric metasurfaces.

## Basic Principles and Various Local Resonances of Dielectric Metasurfaces

Dielectric building blocks can support various local resonances such as ED and MD resonances, which provide a basic capability to locally manipulate EM waves in the manner of resonant photonics. Lattice resonances originating from the collective lattice responses of periodically or quasi-periodically arrayed nanostructures are also basic resonances (Hu et al., 2019; Tang et al., 2019), which can be described by the band theory (Li et al., 2019a). On the other hand, the lattice resonances can also be attributed to local resonances with mutual interparticle inferences (Evlyukhin et al., 2010). In this section, we will discuss basic principles and local EM resonances of dielectric metasurfaces. Compared with traditional optics that mainly relies on optical manipulation by optical path superposition, the abundant resonances enabled by dielectric nanostructures guarantee that the optical fields can be manipulated efficiently in an ultrathin thickness with a high degree of freedom.

### Mie Resonances in Dielectric Nanostructures

**Figure 2:**
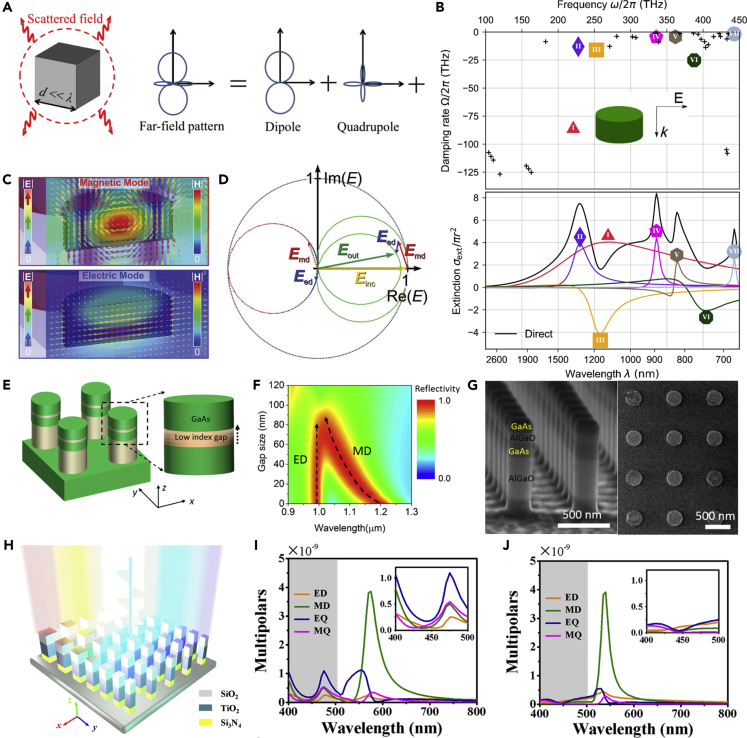
Tailoring the Multipole EM Resonances by Dielectric Resonators (A) Dielectric scatterer with a typical size much smaller than the operating wavelength. The far-field pattern of the scattered light can be decomposed in terms of multipolar field components. (B) Calculated complex frequencies of the resonant modes of the silicon resonator and the contributions of each mode. (C and D) (C) Electric and magnetic field distributions of the EM modes of the Huygens' silicon resonator. (D) The vector diagram depicting the superposition of EM resonances and the incident waves. (E–G) (E) Schematic of the layered Huygens' dielectric resonator. (F) The resonances as a function of the gap size and the wavelength. (G) SEM images of the metasurface. (H–J) (H) Schematic of the multipolar-modulated metasurface. The multipole components for (I) all-TiO_2_ resonators and (J) multi-dielectric resonators. Figures reproduced from: (A) (Campione et al., 2015) Copyright 2015, Optical Society of America; (B) (Powell, 2017) Copyright 2017, American Physical Society; (C and D) (Decker et al., 2015) Copyright 2015, WILEY-VCH; (E–G) (Liu et al., 2017a) Copyright 2017, American Chemical Society; (H–J) (Yang et al., 2019) Copyright 2019, American Chemical Society.

The fundamental principle of dielectric nanostructures to manipulate the optical fields is Mie-type resonances, which is the Mie solution to Maxwell's equations. Generally, the characteristic size of the nanostructures is about *λ*/*n*_eff_, where *λ* is the resonant wavelength and *n*_eff_ is the effective refractive index of the nanostructure. The resonant wavelength and the effective refractive index are both functions of the geometric parameters of the nanostructures. As a result, the scattering properties can be conveniently engineered by tailoring the geometric parameters of meta-atoms. As shown in Figure 1B, the Mie-type scattering of a dielectric resonator contains different components such as ED, MD, electric quadrupole (EQ), and magnetic quadrupole (MQ) resonances (Koshelev and Kivshar, 2020), which enable both near-field and far-field manipulations of EM waves for different functionalities. Figure 2A illustrates the Mie-type scattering of a dielectric cubic scatterer (Campione et al., 2015). The far-field scattering pattern can be decomposed into terms of multipolar field components:

(Equation 1) $$I=\frac{2\omega^{4}}{3c^{2}}{\left|P\right|}^{2}+\frac{2\omega^{4}}{3c^{2}}{\left|M\right|}^{2}+\frac{4\omega^{5}}{3c^{4}}\left(P\cdot T\right)+\frac{2\omega^{6}}{3c^{5}}{\left|T\right|}^{2}+\frac{\omega^{6}}{5c^{5}}\sum {\left|Q_{\alpha \beta }\right|}^{2}+\frac{\omega^{6}}{40c^{5}}\sum {\left|M_{\alpha \beta }\right|}^{2}+...$$

where *P*, *M*, *T*, *Q*_*αβ*_, *M*_*αβ*_ are defined as the ED, MD, toroidal dipole, EQ, and MQ, respectively. These Mie-type resonances can interfere or couple with each other, leading to high degree of freedom to manipulate the scattering properties of dielectric nanostructures. Figure 2B shows the extinction spectrum and the contributions from each of the modes in a silicon disk (Powell, 2017). Different resonance components play different roles in the extinction spectrum, which suggests that one can modulate the scattering properties of dielectric nanostructures by controlling each resonance component. Compared with metallic nanostructures, which can generate ED/MD resonances with nanorods/split-ring resonators (Kim et al., 2018; Wang et al., 2019; Wu et al., 2017), one of the merits of using dielectric nanostructures is that they can combine different EM resonances in a simple single nanostructure. This property enables the dielectric metasurface can be designed as a high-efficiency Huygens' metasurface (Decker et al., 2015). As shown in Figures 2C and 2D, by simultaneously generating MD and ED resonances and overlapping them, the reflection of the metasurface can be significantly suppressed. This phenomenon can also be interpreted by the effective refractive index model. Since electric and magnetic resonances can be simultaneously generated, the effective permittivity and permeability can also be modulated. When the effective permittivity and permeability satisfy the impedance matching condition, the reflection can be minimized. On the other hand, the suppression of reflection can also be interpreted by the Kerker effect (Kerker et al., 1983), that is, the destructive interference in the reflection direction owing to different spatial symmetry of different Mie-type resonances (Limonov et al., 2017).

Dielectric layered structures provide a versatile and efficient method to accurately manipulate each EM resonance component in the dielectric nanostructures. Figure 2E shows a split dielectric III-V nano-resonator containing a GaAs nanostructure with a low index (AlGaO) gap in it. Such a design can accurately tune the MD resonance at different operating wavelengths with different geometric parameters (Liu et al., 2017a). As shown in Figure 2F, when spectrally overlapping the ED and MD resonances and satisfying the first Kerker condition, suppression in reflectivity can be achieved in a broadband region. The scanning electron microscope (SEM) images show that such split layered structures can be well fabricated (Figure 2G). Another triple-layered dielectric structure supporting low Fabry-Pérot quality factor was proposed to generate high-quality structural colors, as shown in Figure 2H (Yang et al., 2019). Compared with all-TiO_2_ nanostructures (Figure 2I), which support significant multipolar Mie-type resonances in the visible region, the triple-layered (SiO_2_-TiO_2_-Si_3_N_4_) resonator can realize suppression of multipolar resonances, especially in the blue light region, leading to a pure reflective spectrum in the whole visible regime (Figure 2J). Such an effects can be employed to realize high-quality filters, structural colors generation, and so on. The capability of dielectric resonators to tailor the multipole EM resonances enables distinguished prospects to manipulate EM fields on demand.

### Other Typical EM Resonances

Based on the versatile manipulation of EM multipole resonances, dielectric metasurfaces can realize abundant typical resonances such as bound states in the continuum (BICs), Fano resonances, and anapole resonances. These resonances enable numerous intriguing applications that are not feasible in conventional optical platforms.

#### Bound States in the Continuum

BICs, which have giant Q factors, were first investigated in quantum mechanics with localized electron waves embedded in the continuous spectrum of propagating waves.

**Figure 3:**
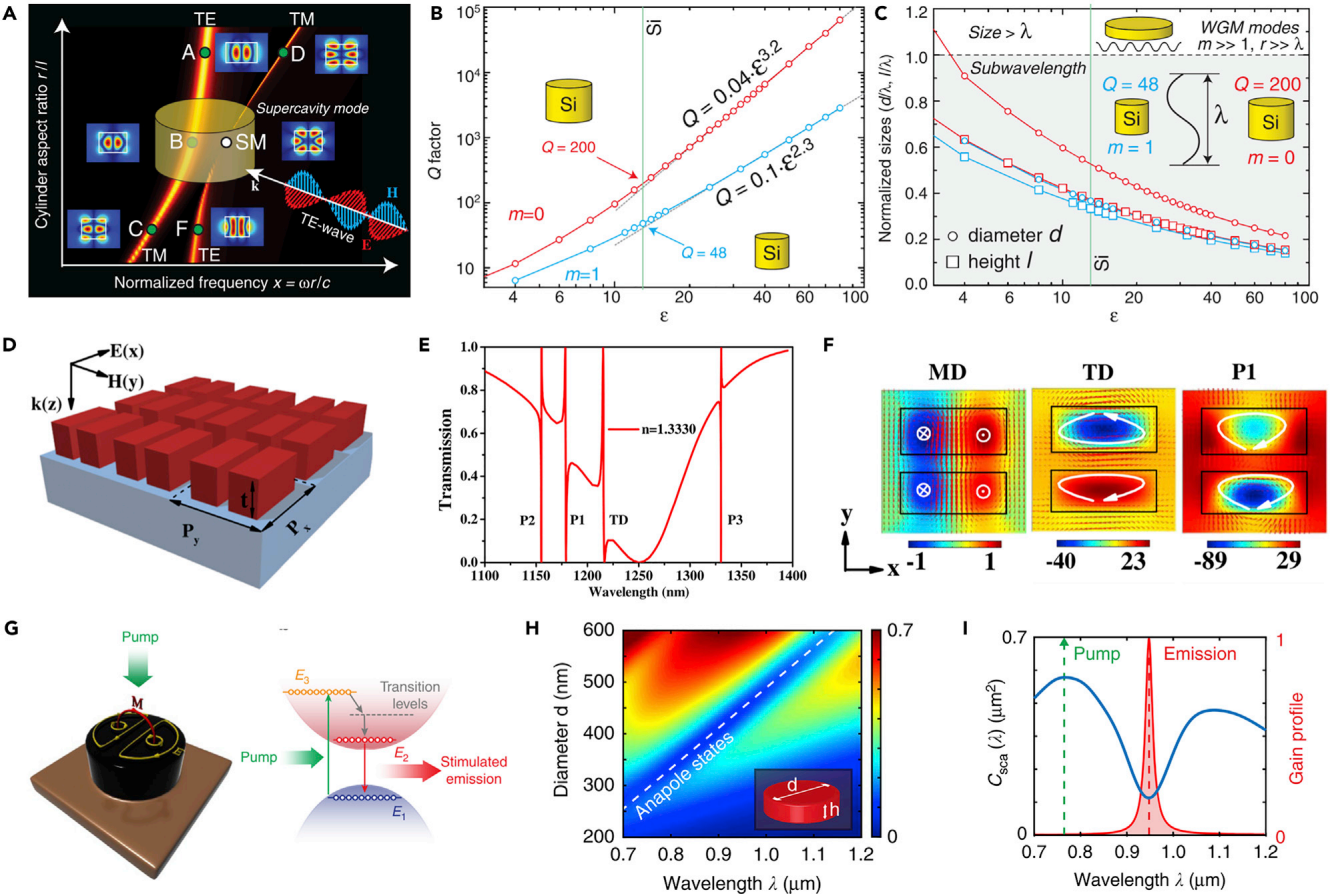
Typical Dielectric Combined Resonances (A–C) (A) Schematic of the BIC resonance and strong mode coupling in the high-index dielectric resonator. (B) Calculated Q factors as a function of the dielectric permittivity. (C) Relative dimensions of the dielectric cylindrical resonator with a super-cavity mode. (D–F) (D) The Fano resonance metasurface is composed of periodic silicon resonator pairs. (E) Calculated transmission showing multiple Fano resonances. (F) EM near-field distributions at different resonant wavelengths. (G–I) (G) Illustration of the anapole nanolaser. The direct bandgap semiconductor is optically pumped leading to stimulated emission. (H) The anapole resonances as a function of the operating wavelengths and the diameter of nanodisk. (I) Scattering cross section for an In_0.15_Ga_0.85_As nanodisk with the Lorentzian gain contained in the scattering suppression region. Figures reproduced from: (A–C) (Rybin et al., 2017) Copyright 2017, American Physical Society; (D–F) (Zhang et al., 2018b) Copyright 2018, Optical Society of America; (G–I) (Totero Gongora et al., 2017) Creative Commons attribution 4.0 International License (http://creativecommons.org/licenses/by/4.0), Copyright 2017, Nature Publishing Group.

BICs in dielectric resonators are realized via modes coupling and destructive interference between different Mie-type resonances. The ideal BICs are not observable and can support infinite Q factors due to the nonradiative property, which means any observable BIC is finite and is quasi-BIC (Gorkunov et al., 2020; Koshelev et al., 2018, 2019; Li et al., 2019c). Figure 3A shows TE (Mie-type) mode and TM (Fabry-Pérot-type) mode strongly interact with avoided crossing behavior at the super-cavity mode (SM) point for a single dielectric cylindrical resonator (Rybin et al., 2017). The SM point with mixed polarization supports unexpectedly high Q factors. As shown in Figure 3B, the calculated Q factors of the SM almost reach 10^5^ for high values of permittivity of the dielectric nanostructures. Specifically, the Q factor is about 200 for Si resonators. Figure 3C shows the relative dimensions of a dielectric cylindrical resonator that supports the super-cavity mode for different sizes of the diameters (circles) and heights (squares). Generally, the larger the dielectric permittivity is, the smaller the spatial relative dimensions of the dielectric resonator will be. This can be attributed to the decrease of the effective resonant wavelength when increasing the permittivity of the resonator.

The symmetry of nanostructures plays an important role in BICs generation. By controlling the mirror symmetry of two zigzag hydrogenated amorphous silicon structures, Huygens' condition and BICs have been bridged, leading to a Huygens' BICs in the near-infrared waveband (Liu and Choi, 2018). By engineering the symmetry properties and the number of the unit cells, quasi-BICs that can significantly boost the nonlinear generation have been realized with a Q factor of 18,511 (Liu et al., 2019c). The second-harmonic generation (SHG) can also be enhanced by more than 3 orders of magnitude by combining the BICs in silicon nanostructures and a WS_2_ monolayer (Bernhardt et al., 2020). Recently, BICs in an individual dielectric resonator rather than in periodic structures have been experimentally demonstrated (Koshelev et al., 2020). By suppressing radiative losses of the nanostructure, the BICs can be generated under an azimuthally polarized vector beam, and the SHG enhancement has been demonstrated.

#### Fano Resonances

Fano resonances in dielectric photonics originate from the interference between a discrete narrow Mie-type state and a continuum state (or a broadband off-resonant state), and the scattering cross section can be described by the Fano formula:

(Equation 2) $$\sigma \left(E\right)=D^{2}\frac{{\left(q+\Omega \right)}^{2}}{1+\Omega^{2}}\text{,}$$

where *q* is the Fano parameter, *Ω* = 2(*E − E*_0_)/*Γ*, *Γ* and *E*_0_ are the resonance width and energy, respectively (Limonov et al., 2017; Miroshnichenko and Kivshar, 2012). Equation (2) can also be applied in different optical spectra, such as transmission, reflection, and scattering in different systems. Figure 3D depicts a dielectric Fano resonator that consists of two dielectric nano-bricks. By elaborate tailoring the periodicity and the gap size of the nano-bricks, multi-band Fano resonances can be generated through the periodical asymmetric paired silicon nano-bricks (Zhang et al., 2018b). The Q factors for P1, P2, and P3 resonances can reach about 2.8×10^4^, 2.9×10^4^, 1.3×10^5^ for the specific design of the asymmetric parameter (Figure 3E). Figure 3F shows the simulated near-field EM distributions for different Mie-type resonances. The ultra-narrow full width at half maximum (FWHM) enables sensitive refractive index sensing of the surrounding materials. By chemically tuning the excitonic states of the perovskite nanostructures, the Fano resonance can be reversibly controlled over a 100 nm frequency range (Tiguntseva et al., 2018).

#### Anapole Resonances

Anapole resonances can be realized by the spectral overlap of different Mie-type resonances. Compared with BICs and Fano resonances, anapole resonances do not require direct coupling between different oscillating modes but undergo spatial mode interferences. As a result, the near-field EM components are significantly enhanced and the far-field scattering is suppressed. With a destructive interference between a toroidal dipole and an out-of-phase oscillating ED, the anapole resonance occurs with vanishing far-field scattering and with strong near-field absorptions (Miroshnichenko et al., 2015). The Q factor of the anapole mode can approach about thirty (Grinblat et al., 2017). Figure 3G illustrates an optically pumped anapole nanolaser realized with a direct bandgap semiconductor (Totero Gongora et al., 2017). Figure 3H shows suppression in the scattering spectra at the anapole resonances for the In_0.15_Ga_0.85_As nanodisk. The anapole resonance gives rise to a remarkably stable steady state, resulting in the generation of an ultrafast pulse of 100 fs (Figure 3I). Taking advantage of the strong enhancement provided by a higher-order anapole mode, efficient third-harmonic generation (THG) can be realized by a single Ge nanodisk (Grinblat et al., 2017). The anapole resonances can also be employed to realize giant photothermal nonlinearity in silicon nanostructures, with a nonlinear index change up to 0.5 for incident light intensity on the order of MW/cm^2^ (Zhang et al., 2020a).

### Nonlinear Resonances

As mentioned above, dielectric nanostructures can boost the generation of nonlinear light taking advantage of strong local interactions between EM waves and the nanostructures (Bar-David and Levy, 2019; Gu et al., 2016; Li et al., 2017a). For example, polarization-dependent control of SHG has been demonstrated with a GaAs metasurface (Löchner et al., 2018). The polarization-dependent property results from the off-diagonal components of the nonlinear susceptibility tensor ($\chi_{ijk}^{\left(2\right)}\neq 0$ for i≠j≠k otherwise zero). A Fano resonance-based GaAs metasurface to realize enhanced SHG can also be achieved by using a symmetry-broken layered nanostructure (Vabishchevich et al., 2018). Strong local resonances such as Fano resonances provide strong nonlinear light-matter interactions and near-field enhancement, which is highly desired in nonlinear conversions (Yang et al., 2015). Since the optical nonlinearity originates from the local nonlinear polarization, either for the bulky medium or for the structured materials, the geometric symmetry of structures can induce the superposition of the local nonlinear polarization. Such effect results in nonlinear P-B phase and the selection rules of harmonic generation (Chen et al., 2014; Li et al., 2015a, 2018b; Tymchenko et al., 2015).

**Figure 4:**
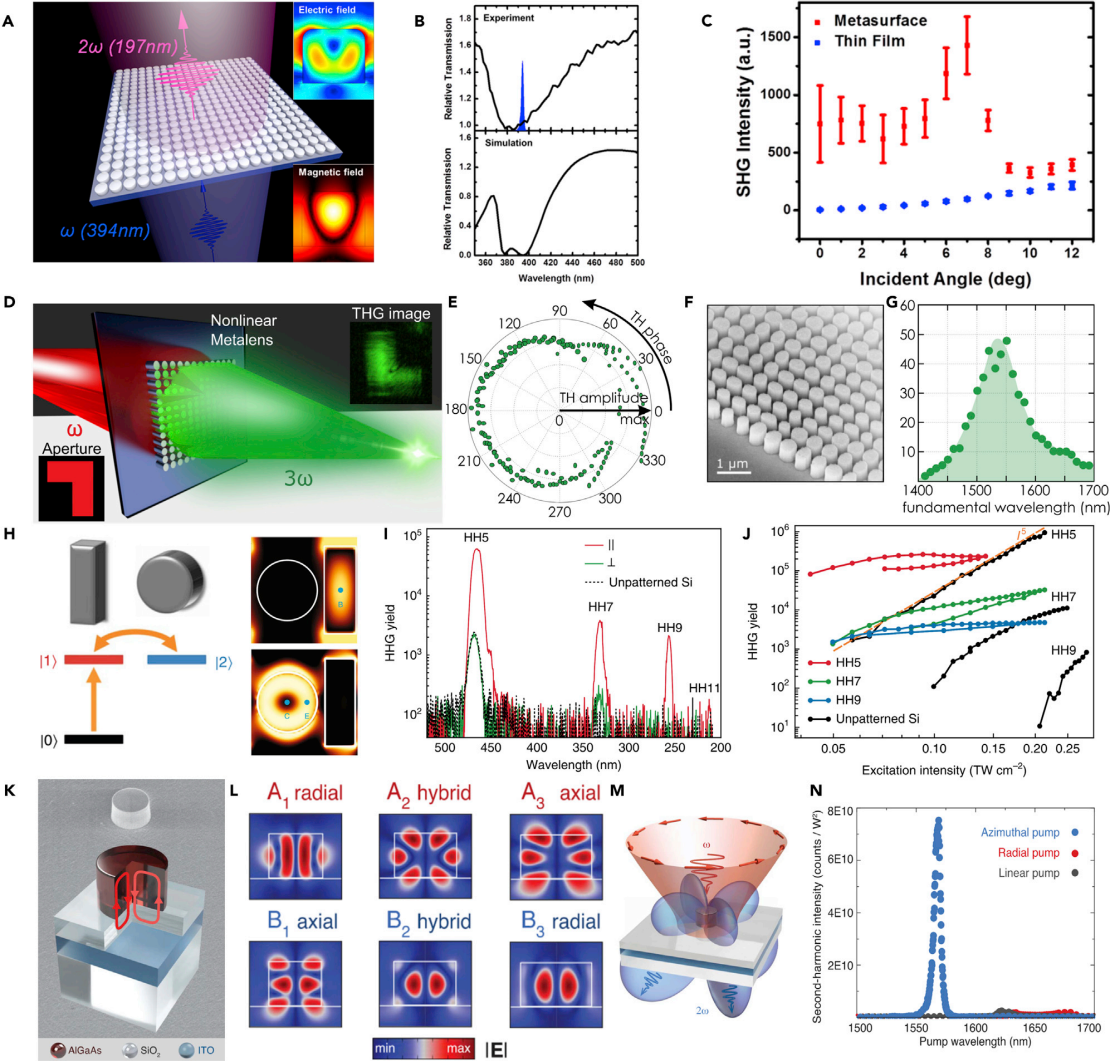
Nonlinear Dielectric Metasurfaces (A–C) (A) UV nonlinear metasurface composed of ZnO resonators. (Insets: calculated electric and magnetic field near the cross section of the nanostructure) (B) Measured and simulated relative transmission spectrum of the nonlinear metasurface. (C) Measured SHG intensity for different incident angles. (D–G) (D) Schematic of the dielectric nonlinear metalens used as an imaging device. (E) Simulated THG amplitude and phase for different nanopillars chosen as the building blocks of the nonlinear metalens. (F) SEM image of the fabricated metalens. (G) Third-harmonic diffraction efficiency as a function of the fundamental illumination wavelength. (H–J) (H) Level scheme for the three-level Fano-resonant system. The “bright” mode in the nano-bar and the “dark” mode in the nano-disk correspond to states |1> and |2>, respectively. (I) High-harmonic spectra of the silicon metasurface. (J) Measured non-perturbative high-harmonic yield as a function of vacuum peak excitation intensity. (K–N) (K) Optical quasi-BIC resonance in an individual dielectric resonator. (L) Simulated near-field EM resonances for different diameters of the resonator. (M) Illustration of the SHG with an azimuthally polarized vector beam as excitation. (N) Measured SH intensity for different incident polarization states. Figures reproduced from: (A–C) (Semmlinger et al., 2018) Copyright 2018, American Chemical Society; (D–G) (Schlickriede et al., 2020) Copyright 2020, American Chemical Society; (H–J) (Liu et al., 2018a) Copyright 2018, Nature Publishing Group; (K–N) (Koshelev et al., 2020) Copyright 2020, American Association for the Advancement of Science.

Basically, the engineering materials decide the working waveband for a nonlinear generation. Researchers employed ZnO nanostructures to realize UV nonlinear metasurfaces and the SHG locates at 197 nm (Figure 4A) (Semmlinger et al., 2018). Figure 4B shows the measured and simulated relative transmission of the nonlinear metasurface with an excitation laser spectrum indicated as the blue area. Compared with a thin ZnO film, the structured metasurface can significantly enhance the SHG intensity by two or three orders of magnitude (Figure 4C). Nonlinear dielectric metasurfaces can also be designed as functional devices, such as nonlinear wavefront control (Wang et al., 2018a) and nonlinear holography (Gao et al., 2018). A nonlinear metalens that can realize nonlinear imaging has been demonstrated (Schlickriede et al., 2020) (Figure 4D). As shown in Figure 4E, the THG phase covers 0−2π range with almost identical amplitude. The SEM image the fabricated metasurface is shown in Figure 4F. For such designs, the nonlinear generation is highly dependent on the resonances of nanostructures, which means the working efficiency is relatively high at the operating wavelengths (Figure 4G). Thus, it is challenging to realize broadband and efficient nonlinear generation. A broadband optical frequency mixer based on GaAs dielectric nanostructures has been realized, which enables SHG, THG, fourth-harmonic generation, sum-frequency generation, two-photon absorption-induced photoluminescence, four-wave mixing, and six-wave mixing simultaneously (Liu et al., 2018b). As a classical analog of electromagnetically induced transparency, enhanced high-harmonic generation was realized based on the Si metasurface (Liu et al., 2018a). As shown in Figure 4H, conversion from state |0 > to state |1 > represents direct excitation of the “bright” mode, and the arrow between state |1> and state |2 > illustrates the coupling in the near-field between the “bright” and the “dark” modes. Figure 4I shows the high-harmonic spectra from polarized excitation pulses. The generated harmonic signal reaches 11th order with a vacuum excitation intensity of 0.071 TW cm^−2^. The measurement is limited by the detection method which introduces some noises. The proposed metasurfaces significantly boost the high-harmonic generation compared with the unpatterned Si layer, especially for weak excitation intesity of the laser (Figure 4J). Recently, an AlGaAs-SiO_2_-ITO hybrid system was proposed to realize BICs generation in an individual resonator and further to enhance the nonlinear optical generation (Figure 4K). By engineering the BICs in an individual dielectric resonator, a total SHG efficiency of 4.8 × 10^−5^ has been achieved (Koshelev et al., 2020). Figure 4L shows the simulated near-field electric distributions for the two modes for different diameters of the resonator. The azimuthally polarized fundamental vector beam excitation is required to realize the BICs in this individual resonator (Figure 4M). The incident polarization states highly affect the SHG intensity, which is consistent with the excitation conditions of the BIC in the nanostructure (Figure 4N).

## Typical Applications of Dielectric Metasurfaces

Based on the versatile capabilities to manipulate EM waves with a high degree of freedom, dielectric metasurfaces can be applied in numerous optical areas, ranging from conventional optical components with miniaturized sizes to intriguing devices that are not feasible with traditional optical designs. In this section, we discuss several typical applications of dielectric metasurfaces that are promising to find potential applications in real life including structural colors, wavefront-shaping, multi-dimensional manipulation of EM waves.

### Structural Colors

**Figure 5:**
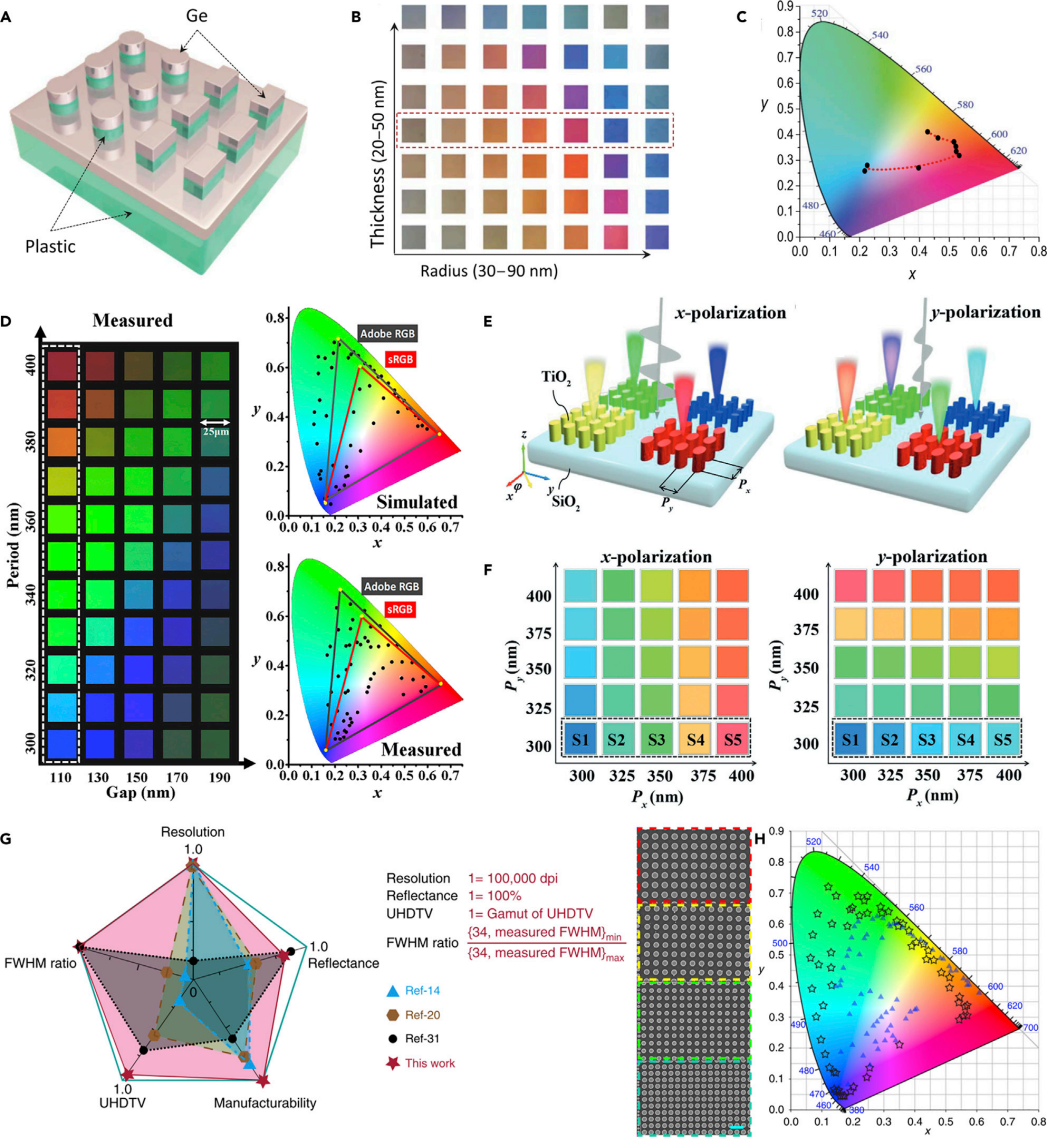
Structural Colors Realized by Dielectric Metasurfaces (A–C) (A) Schematic illustration of the high-index dielectric resonators. (B) Palette of colors for different radii and the deposited Ge film thickness. (C) Gamut loop of laser-printed structural colors covering CMY colors in a standard CIE-1931 color space. (D) Captured color palette and the corresponding CIE-1931 chromaticity coordinates for the ultra-highly saturated structural colors realized by a SiO_2_-TiO_2_-Si_3_N_4_ layered metasurface. (E–F) (E) Illustration of the polarization-sensitive structural colors. (F) Simulated colors for different periodicity along *x*- and *y*-directions. (G–H) (G) The high-performance structural colors generated from a silicon metasurface, which employs a refractive index matching layer. The reflection bandwidth and the background reflection are both reduced, leading to high-performance structural colors compared with other researches. (H) Simulated colors in the CIE-1931 color space. Figures reproduced from: (A–C) (Zhu et al., 2017) Copyright 2017, American Association for the Advancement of Science; (D) (Yang et al., 2019) Copyright 2019, American Chemical Society; (E–F) (Yang et al., 2018) Copyright 2018, WILEY-VCH; (G–H) (Yang et al., 2020) Creative Commons attribution 4.0 International License (http://creativecommons.org/licenses/by/4.0), Copyright 2020, Nature Publishing Group.

Structural colors provide a method to generate colors based on the surface structures of an object rather than by the chemical materials, such as the wings of butterflies (Okada et al., 2013). Taking advantages of local resonances or band gaps in structured designs, the reflectivity can be modulated with a high degree of freedom (Ito et al., 2019; Quan et al., 2020; Raj Shrestha et al., 2015; Sun et al., 2017), especially when the building blocks can be artificially designed. As shown in Figure 5A, a high-index dielectric Ge-based metasurface was proposed to realize structural colors (Zhu et al., 2017). By harvesting from above-bandgap absorption, the Ge nanostructures can be laser-post-processed to manipulate the morphology-dependent resonances in Ge nanostructures, leading to laser-printable structural colors (Figures 5B and 5C). Although Ge is not as lossless as some of the dielectric materials such as titanium dioxide, the intrinsic loss of Ge is comparable to that of aluminum in the visible region. The plasmonic aluminum-based structural colors have also been investigated due to the wide spectral plasmonic band such as a plasmonic color filter (Li et al., 2016) and dual-state micro-image encoding (Heydari et al., 2017). On the other hand, by selecting high-index lossless dielectric materials, high-quality structural colors can be generated by the judicious design of multipole EM resonances. Figure 5D is the experimental color pallet realized by a SiO_2_-TiO_2_-Si_3_N_4_ triple-layered metasurface (Yang et al., 2019). The EM dipole and quadrupole resonances can be subtly tailored by designing the thickness in each layer. Consequently, the FWHM of the reflection spectra can be reduced to about 20 nm, leading to vivid colors across the visible regime. Although theoretically the FWHM can be further optimized and reduced, it is not necessary to do so because the reduction of FWHM will also lower the reflective intensity in real applications. Thus, the design of structures is also a balance between FWHM and efficiency. When the nanostructures are anisotropic, metasurfaces can support different resonances in different directions. As shown in Figures 5E and 5F, for different incident polarization states, the color palettes of elliptic TiO_2_ nanopillars show different reflected colors as varying the lattice sizes along *x*- and *y*-directions, respectively (Yang et al., 2018). The refractive index of silicon is higher than TiO_2_ in the visible region, and silicon is easier to be fabricated. Researchers also take efforts to develop structural colors based on silicon nanostructures (Flauraud et al., 2017; Proust et al., 2016). To overcome the intrinsic loss of amorphous silicon in the visible regime, researchers also developed structural colors based on hydrogenated amorphous silicon (Park et al., 2017) and monocrystalline silicon. On the other hand, by introducing the index matching effect, amorphous silicon nanostructures have also been demonstrated to be capable of printing beyond the sRGB color gamut (Dong et al., 2017). Figures 5G and 5H show a high-performance structural color generation based on amorphous silicon by combining with a refractive index matching layer (Yang et al., 2020). The spatial resolution, manufacturability, efficiency, and FWHM all reach a high level compared with other works. Generally, structural color generation requires tedious simulation, optimization, trade-offs among resolution, color quality, and angular stability. Recently, researchers have developed deep neural networks to generate structural colors from more random nanostructures (Gao et al., 2019). This may put forward more high-performance structural color designs that may find applications in color display and information storage.

### Wavefront-Shaping Designs

Wavefront-shaping enables the EM waves to propagate or transmit to a far distance when carrying the optical information. Generally, the wavefront of EM waves is decided both by the intensity and phase at each spatial point, while phase plays a much more important role as suggested by the famous Gerchberg-Saxton algorithm (Gerchberg and Saxton, 1972). Compared with conventional diffractive elements (CDEs), in which the phase of EM waves is accumulated based on the length of the ray path inside the building materials, metasurfaces can realize phase manipulation by tailoring the local resonances and symmetry of the nanostructures (Banerji et al., 2019; Engelberg and Levy, 2020; Huang et al., 2018). Once the phase accumulation process is achieved, the output light travels according to the diffractive theory for both of the CDE and metasurfaces. To date, there has been no solid demonstration of whether CDE or metasurfaces are more successful or potentially successful. For many applications CDE are sufficient and more cost effective. However, metasurfaces provide more degrees of freedom to engineer the optical fields such as polarization-sensitive functionalities, which is challenging to be incorporated in CDE. On the other hand, for high-numerical-aperture devices, the required minimum feature size reaches subwavelength scale according to the Nyquist theorem, which will break the boundary between CDE and metasurfaces. Dielectric metasurfaces provide an efficient platform to realize wavefront-shaping directly, some of the intriguing applications include metalenses, multifunctional metasurfaces, metasurface holography, and computational metasurfaces.

#### Metalenses

Since metasurfaces can manipulate the phase of the optical fields pixel by pixel, all of the traditional diffraction-based devices can be redesigned with metasurfaces. Taking advantage of the subwavelength properties of nanostructures, dielectric metalenses can realize high-quality focusing and imaging compared with commercial objectives (Khorasaninejad and Capasso, 2017; Khorasaninejad et al., 2016b; Zuo et al., 2018). The phase of nanostructures can be designed according to:

(Equation 3) $$\phi \left(x,y\right)=\frac{2\pi }{\lambda_{d}}\left(f-\sqrt{x^{2}+y^{2}+f^{2}}\right)$$

**Figure 6:**
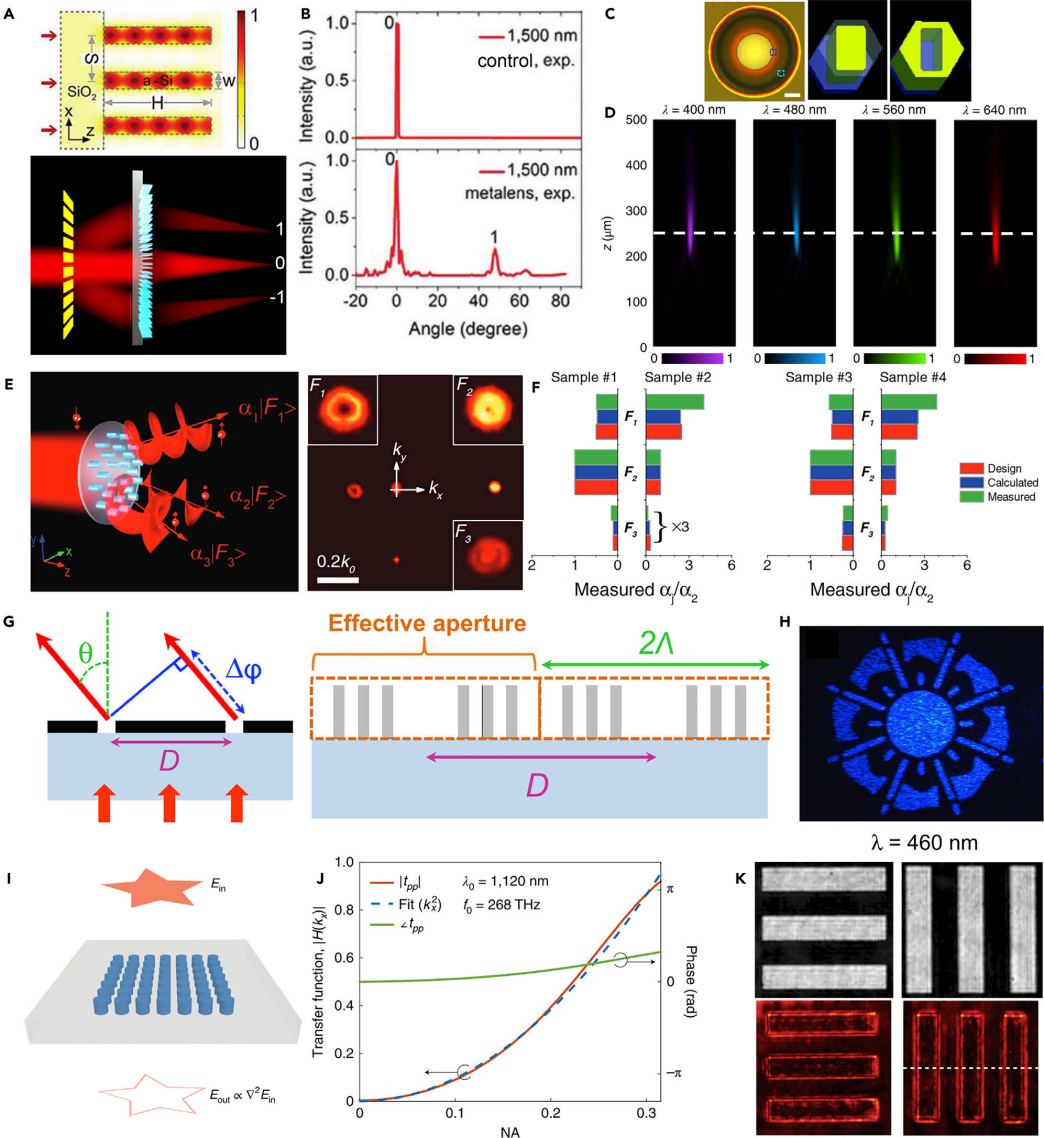
Wavefront-Shaping Applications (A and B) (A) Building blocks of the Fourier metalens and the waveguide modes inside the silicon resonator. The metalens can perform the Fourier transform of the incident light. (B) Comparison between the measured Fourier spectra by the metalens and a commercial Fourier lens. (C and D) (C) An image and the building blocks of the broadband achromatic GaN metalens. (D) Measured focusing profiles for different incident wavelengths. (E and F) (E) A multifunctional metasurface that can accurately distribute different intensity ratios to different working channels without intrinsic noises. (F) Comparison among the designed, calculated, and measured intensity in each channel of different metasurface samples. (G and H) (G) Building blocks of the meta-holograms. The transmitted phase is decided by the arrangement of the nanostructures and is irrelative to the operating wavelengths. (H) The measured hologram image of the device. (I–K) (I) Illustration of the device that can perform second-order derivative, i.e. a Laplacian operator. (J) Optical transfer function and the quadratic fitting for different NA (or working incident angles). (K) Experimental edge detection based on the second-order derivative of an image. (Liu et al., 2018c) Copyright 2018, WILEY-VCH; (C–D) (Wang et al., 2018b) Copyright 2018, Nature Publishing Group; (E–F) (Liu et al., 2019a) Copyright 2019, WILEY-VCH; (G–H) (Khorasaninejad et al., 2016a) Copyright 2016, American Association for the Advancement of Science; (I–K) (Zhou et al., 2020) Copyright 2020, Nature Publishing Group.

However, the chromatic aberrations, which usually limit the imaging quality of an imaging system, are challenging to be overcome in the metasurface platform because the angular and frequency dispersions need to be considered. Learning from conventional lens designs, metalens doublets were proposed to correct monochromatic aberrations (Arbabi et al., 2016; Groever et al., 2017). By employing the propagation phase in silicon micro-waveguides that support weak angle-dispersion resonances, a wide-angle Fourier metalens can be realized (Figure 6A) (Liu et al., 2018c). The spatial frequency spectrum shows that the Fourier metalens can resolve spatial frequency until about 60°, which in comparison cannot be resolved by a commercial Fourier lens (Figure 6B). Recently, researchers also proposed a general strategy to control the angular dispersions by controlling the near-field couplings and radiation patterns (Zhang et al., 2020b). Figure 6C shows an achromatic metalens design that can operate from 400 nm to 660 nm by introducing the phase compensation for different wavelengths using GaN nanostructures (Wang et al., 2018b). The measured focusing intensity profiles at different operating wavelengths show that the proposed GaN metalens is achromatic in a wide waveband in the visible region (Figure 6D). Although different designs to realize achromatic metalenses have been proposed (Chen et al., 2018a; Khorasaninejad et al., 2015, 2017; Wang et al., 2017b), it is still challenging to realize metalenses that can correct all kinds of imaging aberrations with high numerical apertures and high efficiency. Recently, a large-area, high-numerical-aperture, and high-efficiency metalens was proposed based on a topology-optimized strategy (Phan et al., 2019). Based on the current development of metalenses, several metalens-systems can be realized such as a light-field camera (Lin et al., 2019), a full-Stokes polarization camera (Rubin et al., 2019), a single-shot depth sensor (Guo et al., 2019), and an aberration-corrected positioning system (Liu et al., 2020).

#### Multifunctional Metasurfaces

Metasurfaces that can deal with concurrent tasks are multifunctional metasurfaces, such as a metasurface functions as an absorber at one waveband and acts as a polarization converter at another waveband (Cheng et al., 2017). By designing the phase distributions, metasurfaces can also transmit multi-vortex beams carrying different orbital angular momentum (Maguid et al., 2016), and the far-field electric distributions can be calculated as:

(Equation 4) $$E=\sum_{j}^{N}\sum_{v}^{n_{j}}A\left(r_{v}^{\left(j\right)}\right)\exp\left\{ikr_{v}^{\left(j\right)}+i\varphi \left(r_{v}^{\left(j\right)}\right)\right\}\text{,}$$

where $r_{v}^{\left(j\right)}$ represents the positions of *v* nanostructures of the *j*-th function. Generally, the multifunctional metasurface can be realized by phase-only designs like computer-generated holography (Jiang et al., 2018; Maguid et al., 2017). By employing both amplitude and phase manipulations, the multifunctional metasurface can realize multi-vortex beams generation with high accuracy (Liu et al., 2019a; Overvig et al., 2019) (Figure 6E). As shown in Figure 6F, such multifunctional designs do not suffer from intrinsic information loss and do not require iteration operations. By employing a bilayer dielectric metasurface, multiwavelength holograms, multiwavelength waveplates, and polarization-insensitive 3D holograms can also be realized based on amplitude and phase manipulations (Zhou et al., 2019a). With the rapid development of dielectric metasurfaces, more multifunctional metasurfaces can be realized to integrate different optical functions such as angle multiplexed metasurfaces (Kamali et al., 2017).

#### Metasurface Holography

Dielectric metasurfaces also provide a wide platform to realize holography. For example, by employing a Huygens' metasurface design, high-efficiency holograms operating in transmission mode was realized based on the Mie-type ED and MD resonances (Zhao et al., 2016). By using the detour phase (Deng et al., 2018; Min et al., 2016), which originates from the displacement of adjacent elements:

(Equation 5) $$\Delta \phi =\frac{2\pi D}{\lambda }\sin\;\theta \text{,}$$

meta-holograms that are irrelative to the operation wavelengths can be realized (Khorasaninejad et al., 2016a), as shown in Figures 6G and 6H. The wavelength dependence of the phase can be suppressed when satisfying $\theta =\sin^{-1}\left(\lambda /\Lambda \right)$, where Λ is the lattice size of the metasurface. By designing different meta-pixels for different operating wavelengths, one can also realize holograms in a smaller working lattice (Wang et al., 2016). The recent development of holography may also promote the evolution of the metasurface-based holography on a compact size (Makey et al., 2019).

#### Computational Metasurfaces

Metasurfaces can also realize computational components based on the wavefront-shaping. For example, the Laplacian operator on the transmitted electric fields, i.e. $E_{out}\propto \nabla^{2}E_{in}$, can also be expressed as $\left(k_{x}^{2}+k_{y}^{2}\right)E_{in}$ for EM waves (Zhou et al., 2020). As shown in Figures 6I and 6J, when the transmission of the nanostructures follows quadratic profiles for different working numerical apertures, i.e. spatial frequencies, the Laplacian operator can be achieved. Such Laplacian operation can also be applied in edge detection (Figure 6K). Based on a similar principle, the first-order spatial differentiation effect can be achieved at oblique light incidence (Cordaro et al., 2019; Zhou et al., 2019b). Taking advantage of the integral transformation capabilities of dielectric metasurfaces, one can also solve the differential and integro-differential equations (Abdollahramezani et al., 2017). Generally, mathematical operations that can be transformed into transmission or reflection modulation, no matter in real space or momentum space, can be realized by metasurfaces, which provide a promising routine for future computational operations in the photonic platform.

### Polarization and Multi-Dimensional Control of EM Waves

**Figure 7:**
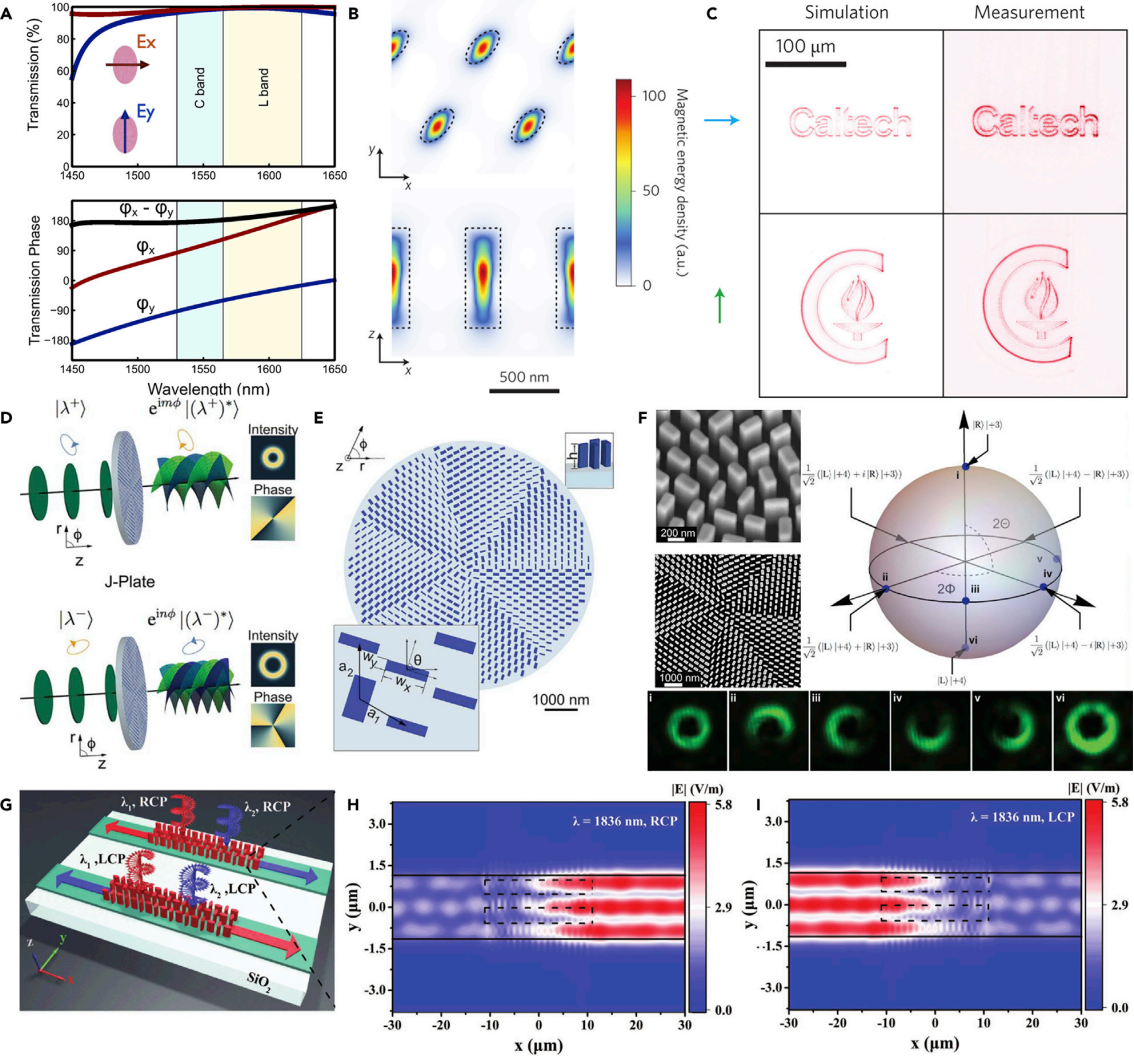
Controlling the Polarization States and Multi-Dimensional Manipulation of Optical Fields (A) Transmission spectra and phase for different incident polarization states. In the C band and L band the device functions as a half-wave plate. (B and C) (B) Silicon nanopillars supporting inside hotspots can be used for complete control of transmitted polarization and phase. (C) Different holograms for different working polarization states. (D–F) (D) Schematic of the arbitrary spin-to-orbital angular momentum conversion. (E) Dielectric nanopillars with different geometric parameters and orientation angles. (F) SEM images and experimental demonstration of the arbitrary combination of two circularly polarized beams with different orbital angular momentum. (G–I) (G) Schematic illustration of the spin-selective and wavelength-selective demultiplexing metasurface. Electric field amplitude distributions for (H) RCP incidence and (I) LCP incidence. Figures reproduced from: (A) (Kruk et al., 2016) Copyright 2016, AIP Publishing; (B–C) (Arbabi et al., 2015) Copyright 2015, Nature Publishing Group; (D–F) (Devlin et al., 2017) Copyright 2017, American Association for the Advancement of Science; (G–I) (Zhang et al., 2019) Copyright 2019, WILEY-VCH.

Polarization manipulation requires anisotropic nanostructures that can interact with EM waves in a different manner for different directions (Chong et al., 2015; Khorasaninejad and Crozier, 2014; Song et al., 2015). As shown in Figure 7A, the elliptic silicon nanopillar can support a superposition of the scattering contributions resulting from electric and magnetic multipolar modes, which realizes high-efficiency phase manipulation in the transmission mode (Kruk et al., 2016). The phase difference for *x*- and *y*-directions remain π in a wide bandwidth, leading to a broadband half-wave plate. Similar designs can also be employed to achieve quarter-wave plates and *q*-plates. Since polarization and phase manipulation can both be conveniently realized by dielectric nanostructures, multi-dimensional manipulation of EM waves becomes possible. Researchers have demonstrated that in high-index and high-aspect-ratio nanostructures, complete and independent control of phase in two orthogonal directions can be realized with the high transmission (Arbabi et al., 2015). By combining the P-B phase (Bomzon et al., 2001; Chen et al., 2012), complete and independent control of phase and polarization can be realized. As shown in Figure 7B, such intriguing properties can be attributed to the magnetic hotspot inside the nanopillars, which is highly sensitive to the geometric parameters of the nanopillars. Based on the complete manipulation of phase and polarization, one can realize several novel applications such as different holograms for different operation directions (Figure 7C). Such design enables information transmission under two orthogonal polarization states, such as chiral and independent holograms in two circular polarization states (Balthasar Mueller et al., 2017). Multi-dimensional manipulation of phase and polarization also provides possibilities for novel optical communications. As shown in Figures 7D and 7E, the designed metasurfaces can convert LCP and RCP polarization states into ones with independent orbital angular momentum. Besides circularly polarized light, similar operations can also be achieved for elliptically polarized light. Figure 7F shows the demonstration of mapping from circular polarizations to two beams with different values of orbital angular momentum in the higher order Poincaré sphere. A waveguide-integrated dielectric metasurface was also realized to achieve spin-selective and wavelength-selective demultiplexing (Zhang et al., 2019). As shown in Figure 7G, the propagation directions of light can be easily controlled by the helicity and wavelength of the incident light, which can be attributed to the P-B phase and phase-matching condition among the couplers. Figures 7H and 7I depict the simulated near-field electric amplitude for different incident polarization states, which demonstrate the spin-selective properties of the metasurface empowered waveguide.

## Tuning the Functions of Dielectric Metasurfaces

Dynamically tuning of metasurfaces remains a challenge during the past years in the visible and near-infrared waveband. One of the reasons lie in the tiny size of the building blocks, which are hard to be dynamically manipulated pixel by pixel. For example, laser tuning provides a promising method to dynamically controlling the geometry of artificial structures (Ling et al., 2020), but the beam size is limited by the diffraction limit. Another reason is that new materials or new strategies are required to alter the refractive index or other key parameters of dielectric materials. To date, researchers have endeavored to find different methods to realize dynamic tuning of metasurfaces, such as using electrical, magneto-optical, chemical, mechanical, and photocarrier-excitation methods (Shaltout et al., 2019b). In this section, we discuss some of the methods that are promising for dynamically tuning the dielectric metasurfaces, including phase-change materials, environmental-tuning configurations, and introduction of other optical dimensions.

### Phase-Change Materials

**Figure 8:**
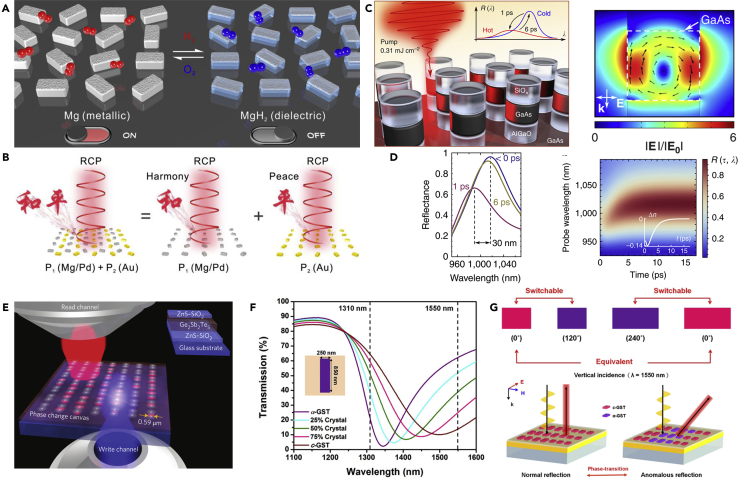
Dielectric Phase-Change Material to Realized Reconfigurable Functions (A and B) (A) Schematic illustration of the hydrogen-responsive metasurface. With hydrogenation (10% H_2_) and dehydrogenation (20% O_2_), the nanostructure can switch between dielectric and metallic states. (B) Two holographic patterns with the “Harmony” pattern being dynamically tuned. (C and D) (C) The Mie-type MD resonance and the fast tuning GaAs metasurface within a tuning window of 6 ps. (D) Reflection spectra of the metasurface at different pump-probe delays and the transient reflectance spectra showing the ultrafast modulation. (E) The “write” channel by trains of femtosecond pulses and the “read” channel in a reconfigurable metasurface. (F and G) (F) By changing the crystallization fraction, the transmission of GST nanorod can be tuned in the infrared regime. (G) By controlling the phase-transition of the GST resonator, the reflective phase can be tuned to realize a phase-gradient metasurface. Figures reproduced from: (A and B) (Li et al., 2018a) Copyright 2018, American Association for the Advancement of Science; (C and D) (Shcherbakov et al., 2017) Creative Commons attribution 4.0 International License (http://creativecommons.org/licenses/by/4.0), Copyright 2017, Nature Publishing Group; (E) (Wang et al., 2015) Copyright 2015, Nature Publishing Group; (F and G) (Chu et al., 2016) Copyright 2016, WILEY-VCH.

Phase-change medium enables direct manipulation of the refractive index of the building blocks of metasurfaces (Chu et al., 2016; Tiguntseva et al., 2018). As shown in Figure 8A, the nanorods can be chemically changed between metallic and dielectric states (Li et al., 2018a). Consequently, the optical resonances of the nanostructures also vary, leading to the dynamic switching of metasurface holograms (Figure 8B). The phase change of Mg nanostructures is invertible, and can also be applied to realize applications such as a color display (Duan and Liu, 2018) and dynamic Janus metasurface (Yu et al., 2018). The GaAs metasurfaces can be tuned with femtosecond laser pulses (Shcherbakov et al., 2017). As shown in Figures 8C and 8D, the free-carrier-induced absolute reflectance modulation of metasurface can reach up to 0.35 within several picoseconds, and the spectral shift of the MD resonance reaches 30 nm. Another promising phase-change material is germanium antimony telluride (Ge_2_Sb_2_Te_5_, GST), which has a crystallization temperature at about 160°C and a melting temperature of about 600°C (Dong et al., 2018; Gholipour et al., 2013; Qu et al., 2018; Sreekanth et al., 2018). Figure 8E illustrates a reconfigurable GST metasurface, with the refractive index change induced by femtosecond pulses (Wang et al., 2015). The written pattern can also be erased by the same laser with different illumination conditions. Based on the proposed platform, researchers have realized binary and grayscale photonic devices and multiplexing focusing devices. By changing the crystallization fractions of GST nanostructures, the transmission spectra can be dynamically controlled (Figure 8F) and the reflective phase can also be switched (Figure 8G), leading to wavefront steering of GST metasurfaces (Chu et al., 2016). Other phase-change materials such as vanadium dioxide, which has the insulator-to-metal transition at a temperature of about 67°C (Kim et al., 2019; Liu et al., 2012), were also investigated to realize tunable metasurfaces. Although plenty of materials can be optically, magnetically, thermally, or chemically induced to realize phase change, several challenges that limit the corresponding applications remain. For example, the dielectric materials are challenging to be dynamically tuned pixel by pixel in the visible and near-infrared region. With the rapid development of material science and metasurfaces, researchers may find more and more promising phase-change materials to realize tunable metasurfaces, at least applicable in some specific scenarios.

### Environmental-Tuning Configurations

**Figure 9:**
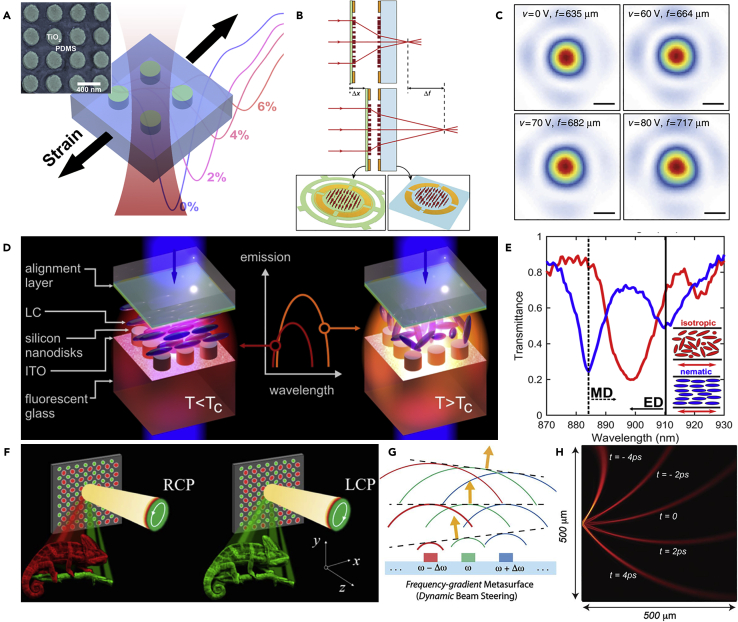
Other Typical Methods to Tune the Dielectric Metasurface (A) Mechanically tuning the transmission spectra by controlling the strain added along the substrate of the metasurface. (B and C) (B) The MEMS tunable dielectric metalens that can rapidly vary the focal length. The scanning frequency can potentially reach several kHz. (C) The focusing intensity distributions at different actuation voltages. (D and E) (D) Active tuning of spontaneous emission by combining dielectric resonators and the liquid crystal cell. (E) The transmission spectra of the metasurface for the nematic state (32°C, blue) and isotropic state (67°C, red). (F) Polarization-controlled color hologram to realize color tuning of metasurfaces. (G and H) (G) Illustration of the frequency-gradient metasurface that can realize ultra-fast continuous dynamic beam steering. (H) Beam distributions at different time instants. Figures reproduced from: (A) (Gutruf et al., 2016) Copyright 2016, American Chemical Society; (B and C) (Arbabi et al., 2018) Creative Commons attribution 4.0 International License (http://creativecommons.org/licenses/by/4.0), Copyright 2018, Nature Publishing Group; (D and E) (Bohn et al., 2018) Copyright 2018, American Chemical Society; (F) (Wang et al., 2017a) Copyright 2017, Optical Society of America; (G and H) (Shaltout et al., 2019a) Copyright 2019, American Association for the Advancement of Science.

Another widely investigated method to tune the functions of dielectric metasurfaces is by changing the operating environments rather than changing the building blocks directly. For example, functions that rely on the lattice sizes of metasurfaces can be tuned mechanically by fabricating the metasurfaces on a stretchable substrate (Malek et al., 2017). As shown in Figure 9A, the transmission spectra can be manipulated when putting a strain along the surface of the polydimethylsiloxane substrate (Gutruf et al., 2016). Since the strain is not easy to be locally controlled, the functions of such kinds of metasurfaces cannot be locally manipulated. Figure 9B depicts another method to realize the dynamic tuning of dielectric metasurfaces based on a micro-electro-mechanical system (MEMS). Such a system controls the distance between two metalenses, and a small change in the distance such as 1 μm may cause a much larger change in the focal distance (about 35 μm) (Arbabi et al., 2018). The focusing profiles for different focal lengths show that the focusing quality is maintained during the dynamic control (Figure 9C). The MEMS method is promising in such a zooming system, and the scanning frequency can potentially reach about kHz. The MEMS method has also been applied in logic operations with the multiple-input-output states (Manjappa et al., 2018).

Recently, liquid crystals as a birefringent complex fluid have gathered much attention due to the capabilities to modify the refractive index map and further to modify the amplitude and phase of the transmitted EM waves (Lininger et al., 2020). As shown in Figures 9D and 9E, when the liquid crystal is heated, the state of the liquid crystal is changed from nematic to isotropic (Bohn et al., 2018), leading to the dynamic tuning of spontaneous emission of the dielectric metasurfaces in the near-infrared waveband. Lately, a one-dimensional phase-only dielectric metasurface has been proposed (Li et al., 2019b). Phase modulation can be achieved based on the liquid crystal layer and dielectric nanostructures, and the thickness of the liquid crystal layer is significantly reduced compared with traditional spatial light modulators. The efficiency of the phase modulation reaches 35%. Since liquid crystals can be locally modulated pixel by pixel, this method provides a promising possibility for the real dynamic and fast tuning of dielectric metasurfaces.

### Introducing Other Optical Dimensions

The functions of dielectric metasurfaces can also be dynamically tuned by introducing other optical dimensions. Here, the term “optical dimension” refers to any variable in the optical fields that can be used to alter the information channel, such as amplitude, polarization state, incident angle, or the operating wavelength. For example, a metalens usually possesses different focal lengths at different operating wavelengths, which leads to imaging of different cut-planes of the 3D object when dynamically tuning the operating wavelengths (Chen et al., 2019). This method employs the spectral dispersion as a new dimension to realize dynamical tuning of focal lengths, and this spectral tomographic method is also ultrafast. Recently, researchers also proposed other methods to alter the functions of metasurfaces using different optical dimensions. As shown in Figure 9F, the phase and polarization states can both be manipulated by the dielectric nanostructures (Wang et al., 2017a). With two incident beams of 632.8 nm and 532 nm, the reconstructed holograms show different colors for different incident polarization states, and the transition color states between the two states can also be realized when continuously changing the incident polarization states. Compared with multifunctional metasurfaces (Shi et al., 2018; Yue et al., 2017; Zhao et al., 2017), such design employs other optical dimensions to manipulate the functions of metasurfaces rather than to serve as functional channels. The dynamic tuning speed is decided by the speed to control the corresponding optical dimensions. Figure 9G shows a design to realize spatiotemporal light control (Shaltout et al., 2019a). The metasurface consists of a group of frequency-gradient nanostructures. Compared with conventional phase-gradient metasurface whose wavefront can be locally modulated, the frequency-gradient metasurface naturally keeps reorienting the wavefront fast. The beam steering angle can be continuously changed to 25° within 8 picoseconds, which is really hard to be achieved with traditional methods (Figure 9H). Such kind of method to alter the functions of dielectric metasurfaces provides a new routine for future tunable metasurfaces.

## Conclusion

In summary, based on the versatile capabilities of dielectric nanostructures to generate abundant high-efficiency resonances, dielectric metasurfaces have been demonstrated to be a promising platform for efficient EM waves manipulations. Although the resonance-based optical components natively possess narrower bandwidth compared with non-resonance-based ones such as diffractive elements, the bandwidth of resonance-based metasurfaces can be significantly broadened by elaborate designs, such as by bringing in multi-band resonances (Aieta et al., 2015) or by imposing specific arrangement of nanostructures (Liu et al., 2017b). It is true that the narrow resonance bandwidth is a drawback for some specific applications. The resonance-based and non-resonance-based designs should be delicately combined together depending on the application scenarios. Herein, we have reviewed the local resonances enabled by dielectric nanostructures. By tailoring the geometric parameters of the resonators, the superposition and scattering properties of multipole EM resonances can be tailored on demand, realizing Huygens' or ultra-narrow band metasurfaces. Ultrahigh Q factor resonances can also be achieved to boost nonlinear generation enhancement. Taking advantage of the EM resonances of dielectric nanostructures, plenty of photonic applications can be achieved such as wavefront-shaping, metalenses, multifunction-integration, holography, and computational metasurfaces. To date, dielectric metasurfaces have been widely investigated ranging from the light source, information transmission to optical detection. We also have reviewed several strategies to dynamically tune the functions of dielectric metasurfaces, including employing phase-change materials, changing the operating environment, and introducing other optical dimensions. We believe the rapid development in fundamental researches and fabrication techniques will enable much more intriguing progresses and approaches for future integrated meta-optics on a compact size.

We envision several directions in dielectric metasurfaces that may have a profound impact on future photonic nano-systems.

(1) Ultra-fast tuning of dielectric metasurfaces.

Although dielectric metasurfaces can be dynamically tuned via numerous methods, the ultra-fast tuning of dielectric metasurfaces pixel by pixel is still challenging. In the gigahertz waveband, metasurfaces can be electrically manipulated through a field-programmable gate array (FPGA) controller (Dai et al., 2018; Zhang et al., 2018a), realizing programmable metasurfaces to arbitrarily control the wavefront. To date, it is challenging to realize similar operations in the visible and near-infrared region due to the lack of efficient operating strategy. On the other hand, the size of the building blocks in these wavebands is about hundreds of nanometers, which is difficult to be manipulated pixel by pixel.

(2) Artificial intelligence empowered designs

The deep learning method has been investigated and applied in various research fields to relieve human beings from tedious designing and optimization works (Liu et al., 2018d; Wang et al., 2020; Yao et al., 2019). Recent developments based on artificial intelligence have enabled metasurface designs with self-training (Liu et al., 2019b), which is highly desired for large-scale designs such as integrated multifunctional metasurfaces. On the other hand, artificial intelligence also provides a possibility to discover physical concepts (Iten et al., 2020) and to discover new design strategies (Liu et al., 2019d; Phan et al., 2019). With the rapid development in artificial intelligence and photonics, these two research fields will combine more and more tightly to promote future high-performance devices.

(3) Integrated dielectric multifunctional meta-systems.

Dielectric metasurfaces are compatible with traditional complementary metal-oxide-semiconductor platforms and are promising to achieve on-chip integrated devices. Combined with the development of structured waveguide and microcavity (Li et al., 2017b; Skryabin et al., 2017), dielectric metasurfaces may integrate different functions together and realize an integrated platform, which can pave the way for future integrated meta-systems supporting mass production in real applications.

(4) Hybrid meta-systems on an extreme size.

As the decreasing of the geometric size of the nanostructures, some intriguing phenomena occur beyond conventional EM theory. For example, a metal-dielectric layered quantum well can boost giant optical nonlinearity with power efficiency of SHG reaching 10^−4^ at an incident pulse intensity of 10 GW/cm^2^ (Qian et al., 2019). New physical principles will dominate when the geometric size of the resonators is small enough (Bekenstein et al., 2020). Although the fabrications techniques nowadays limit the realization of metasurfaces on an extreme size, the underneath intriguing physics will promote more and more researchers to open up the new era.
